# Effects of Switching from Stavudine to Raltegravir on Subcutaneous Adipose Tissue in HIV-Infected Patients with HIV/HAART-Associated Lipodystrophy Syndrome (HALS). A Clinical and Molecular Study

**DOI:** 10.1371/journal.pone.0089088

**Published:** 2014-02-26

**Authors:** Pere Domingo, María del Mar Gutierrez, José Miguel Gallego-Escuredo, Ferran Torres, Gracia María Mateo, Joan Villarroya, Ignacio de los Santos, Joan Carles Domingo, Francesc Villarroya, Luis Del Rio, Vicente Estrada, Marta Giralt

**Affiliations:** 1 Infectious Diseases Unit, Hospital de la Santa Creu i Sant Pau, Universitat Autònoma de Barcelona, Barcelona, Spain; 2 Department of Biochemistry and Molecular Biology and Institute of Biomedicine, University of Barcelona, Barcelona, Spain; 3 Biostatistics and Data Management Platform, Institut d′Investigacions Biomèdiques August Pi i Sunyer (IDIBAPS), Hospital Clinic, Biostatistics Unit, School of Medicine, Universitat Autònoma de Barcelona, Barcelona, Spain; 4 Institut de Recerca del Hospital de la Santa Creu i Sant Pau, Barcelona, Spain; 5 Infectious Diseases Unit, Hospital de la Princesa, Madrid, Spain; 6 CETIR, Barcelona, Spain; 7 Infectious Diseases Unit, Hospital Clínico de San Carlos, Madrid, Spain; 8 Institute of Biomedicine, University of Barcelona, Barcelona, Spain; 9 Fisiopatología de la Obesidad y Nutrición, Centros de Investigación Biomédica en Red (CIBER), Santiago de Compostela, Spain; Rush University, United States of America

## Abstract

HIV-1/HAART-associated lipodystrophy syndrome (HALS) has been associated with exposure to stavudine (d4T) through mitochondrial dysfunction. We performed a 48-week study to assess the effects of switching from d4T to raltegravir (RAL) on metabolic and fat molecular parameters of patients with HALS. Forty-two patients with HALS and a median exposure to d4T > 7 years were switched to RAL and followed for 48 weeks. Fasting metabolic tests, HIV RNA, CD4 cell count, and fat measured by DEXA were obtained at baseline and week 48. mtDNA and gene transcripts for PPAR gamma, adiponectin, cytochrome b, Cox IV, TNF alpha, MCP-1 and CD68 were assessed in paired subcutaneous fat tissue biopsies. Lipid parameters, fasting glucose, insulin, and HOMA-IR did not change significantly. Whole body fat (P = 0.0027) and limb fat mass (P<0.0001) increased from baseline. Trunk/limb fat ratio (P = 0.0022), fat mass ratio (P = 0.0020), fat mass index (P = 0.0011) and percent leg fat normalized to BMI (P<0.0001) improved after 48 weeks. Relative abundance of mtDNA, expression of PPAR gamma, adiponectin, Cyt b, and MCP-1 genes increased, whereas Cox IV, TNF alpha, and CD68 did not change significantly from baseline. Switching from d4T to RAL in patients with HALS is associated with an increase in limb fat mass and an improvement in markers of adipocyte differentiation and mitochondrial function in SAT.

## Introduction

The landscape of HIV-1 infection has been changed forever by the availability, in developed countries, of highly active antiretroviral therapy (HAART) since 1996. However, the doubtless efficacy of HAART is still shadowed by significant toxicity, especially long-term toxicity [1]. Among antiretroviral drug-associated toxicity, that associated with the use of nucleoside reverse transcriptase inhibitors (NRTI) stands out [2]. HIV-1/HAART-associated lipodystrophy syndrome (HALS) is one of the most severe adverse effects related to NRTI use, especially with stavudine (d4T) for which there is overwhelming epidemiological evidence linking its use to the development of HALS [1], [3].

Management strategies for HALS have included diet modifications, switches from NRTI to other drugs and pharmacological interventions [1], [3]. However, the results for most of these strategies have been quite disappointing in terms of reversing lipoatrophy [1], [3]. Among partially successful strategies, switching between NRTI, especially from thymidine analogues to abacavir (ABC) or tenofovir (TDF) has been associated with significant limb fat gains [4], [5].

Raltegravir (RAL) is the first integrase inhibitor and it has exhibited a benign safety profile with no known deleterious effects on fat content in clinical trials [6]. We measured fat content changes over time in virologically controlled HIV-1-infected patients switching from d4T to RAL. In addition, in a subgroup of patients, changes in expression of molecular markers in subcutaneous adipose tissue (SAT) level were also studied. Our working hypothesis was that such a change would induce an increase in subcutaneous fat and an improvement in molecular parameters in SAT in these patients.

## Patients and Methods

### Subjects

All patients for this observational study were recruited through three HIV-1 infection clinics, between July 2008 and December 2009, at the *Hospital de la Santa Creu I Sant Pau* in Barcelona, and *Hospital Clínico* and *Hospital de la Princesa*, the latter two in Madrid. They attend a population of 3800 HIV-1-infected patients on active follow-up. Patients recruited for this study were those with HIV infection, treated with a d4T-based antiretroviral regimen who had clinically-evident HALS. Exclusion criteria included previous use of RAL, including failure with a RAL-based regimen, and the absence of HALS. At baseline, d4T was switched to RAL whereas the other two drugs of the regimen remained unchanged until week 48.

At the time of study entry no patient used any other drug known to influence glucose metabolism or fat distribution such as anabolic hormones or systemic corticosteroids, uridine, recombinant human growth hormone, or appetite stimulants. The diagnosis of AIDS was based on the 1993 revised case definition of the Centers for Disease Control and Prevention [7]. Written informed consent was obtained from the patients at study entry. The study was approved by the Ethics Committees of the Hospital de la Santa Creu i Sant Pau, Hospital Clínico de San Carlos and Hospital de la Princesa.

### Body composition measurements

Subjects were weighed on calibrated scales after removing shoes, outdoor clothing, and other heavy items. Body mass index (BMI) was calculated by dividing the weight in kilograms by the square of the height in meters. Waist circumference was measured to the nearest millimeter using anatomical landmarks as defined for the Third National Health and Nutrition Evaluation Survey [8].

Whole body dual energy X-ray absorptiometry (DEXA) scans (Hologic QDR-4500A Hologic, Inc, 590 Lincoln St, Waltham, MA 02154, USA) were conducted by a single operator at each site on all the patients. Instruments were calibrated daily. The percentage fat at the arms, legs and central abdomen (calculated from the mass of fat versus lean and bone mass) as well as total lean body mass in kilograms was recorded. All DEXA scans were centrally read by the same evaluator (LDR, CETIR), blind to patient therapy. We measured fat symmetry distribution, the following fat ratios were analyzed, trunk/limb fat ratio by dividing trunk fat by limb fat [1], fat mass ratio by dividing the percentage of trunk fat by the percentage of lower limb fat [9], fat mass index by dividing whole body fat by squared height in meters [10], and leg fat percentage normalized to BMI by dividing the percentage of leg fat mass by BMI [11].

### Definition of HALS and metabolic syndrome

Lipoatrophy was identified by patient self-reporting and/or clinician observation of relevant changes to the face (loss of cheek and/or preauricular fat pads), arms, legs, and subcutaneous abdominal tissue, and confirmed clinically by qualitative physical examination. Lipohypertrophy was diagnosed by the presence of central adiposity which was defined by a waist-hip ratio (WHR) of >0.90 in men, and >0.80 in women) [8], breast enlargement in women or the appearance of cervical fat pads or supraclavicular fat accumulation [12]. Both clinical situations occurred in an isolated manner or concomitantly and any combination of them was considered to be a mixed syndrome.

Visual aspects of HALS were assessed with the Lipodystrophy Severity Grading Scale (LSGS) based on that reported by Lichtenstein et al. [12]. In addition, facial lipoatrophy was also assessed through the scale of Fontdevila et al. [13] which established four degrees of facial lipoatrophy ranging from normal to grade three (or severe). A severe loss of SAT was defined as baseline limb fat <3 kg. The metabolic syndrome was defined according to the U.S. National Cholesterol Education Program (NCEP) Adult Treatment Panel III Guidelines [14] and modified as recommended in the latest American Heart Association/National Heart, Lung, and Blood Institute Scientific Statement [15].

### Biochemistry laboratory measurements

All laboratory investigations were performed after a 12 hour overnight fast and at least 15 minutes after the placement of a peripheral intravenous catheter. Patients were seated during blood drawing and abstained from smoking at least 15 minutes before sampling; tourniquet use was avoided when possible and, if not, was maintained for less than 1 minute. All lipid measurements were performed in a Hitachi 911 system from Roche (Basel, Switzerland). Serum total cholesterol and triglycerides were measured by fully enzymatic standard method and high-density lipoprotein (HDL) cholesterol by a direct method using polyethylene glycol modified enzymes (PEGME) [16]. Low density lipoprotein (LDL) cholesterol was measured after ultracentrifugation according to the Lipid Research Clinic recommended method, but utilizing the PEGME method for HDL cholesterol instead of precipitation. Insulin resistance was estimated by the homeostasis model assessment method (HOMA-IR) as the product of the fasting concentrations of plasma insulin (µunits/ml) and plasma glucose (mmol/l) divided by 22.5 [17].

### Fat tissue samples, mitochondrial DNA and gene expression studies in SAT

Fat tissue samples were retrieved at baseline and 48 weeks after switching. They were obtained from SAT abdominal depot through a small surgical biopsy performed by an 8 mm punch under local anesthesia with mepivacaine. The SAT obtained was immediately frozen in liquid nitrogen and stored at −80°C until RNA extraction (see below). The amount of mitochondrial DNA was determined as previously reported [18] and expressed as the relative amounts of mtDNA per nuclear DNA. The expression of gene transcripts encoding for proteins relevant to adipocyte biology and function was assessed: peroxisome proliferator-activated receptor gamma (PPAR gamma), and adiponectin to explore adipocyte differentiation, Cyt b and cytochrome oxidase subunit IV (Cox IV) to explore mitochondrial toxicity, and tumor necrosis factor alpha (TNF alpha), monocyte chemotactic protein-1 (MCP-1) and CD68 (a signal of macrophage infiltration) to explore inflammation. After homogenization of tissue samples in RNeasy lysis buffer (Qiagen, Hilden, Germany), RNA was isolated using a column-affinity based methodology that included on-column DNA digestion (RNeasy; Qiagen). One microgram of RNA was transcribed into cDNA using MultiScribe reverse transcriptase and random-hexamer primers (TaqMan Reverse Transcription Reagents; Applied Biosystems, Foster City, California, CA, USA). For quantitative mRNA expression analysis, TaqMan reverse transcriptase (RT)-polymerase chain reaction (PCR) was performed on the ABI PRISM 7700HT sequence detection system (Applied Biosystems). The TaqMan RT-PCR reactions were performed in a final volume of 25 µl using TaqMan Universal PCR Master Mix, No AmpErase UNG reagent and specific TaqMan primer pair probes: PPAR gamma, Hs00234592; adiponectin, Hs00605917; cytochrome b, Hs02596867; COXIV, Hs00266371; TNF alpha, Hs00174128; MCP-1, Hs00234140; CD68, Hs00154355; RPLO, Hs9999902. Controls with no RNA, primers, or RT were included in each set of experiments. Each sample was run in duplicate, and the mean value of the duplicate was used to calculate the mRNA levels for the genes of interest. Values were normalized to that of the reference control (RPLO) using the comparative 2-ΔCT method, following the manufacturer's instructions. Parallel calculations performed using the reference gene HPRT (Hs99999909) yielded essentially the same results.

### Statistical analyses

Data are expressed as median with inter-quartile range (IQR) or as frequencies and percentages or otherwise specified. Continuous variables were assessed with the Wilcoxon signed-rank test and the Mann-Whitney test for dependent and independent data, respectively. Categorical data were compared by use of the Fisher's exact test. Differences between baseline and 48-week data were assessed with paired t test. The level of significance was established at 0.05 and all reported P values are two-sided. All analyses were performed with the SAS version 9.2 software (SAS Institute Inc., Cary, NC). A logistic regression analysis was used to examine the association of baseline parameters with limb fat gain ≥800 grams over 48 weeks; variables associated with a P<0.1 in the bivariate analyses were included in the multivariate stepwise analysis.

## Results

### Population studied and viro-immunological status

Forty-two Caucasian patients were recruited. There were 32 men (76.2%) and 10 women (23.8%), with a mean age of 45.3±7.43 years (median: 45.6 years [IQR: 41.5–50.0]). Demographic, viro-immunological, metabolic and fat parameters of these patients were not different with respect to recruitment center. Prior AIDS-defining conditions were present in more than 60% of the patients. The demographics, virological and immunological parameters are shown in table 1. Most of the patients (38, 90.5%) had undetectable viral load at baseline, and among those with detectable viral load, the median was 148 (IQR: 70–288) copies/ml. At week 48, 30 (71.4%) patients had undetectable viral load (OR = 2.53; 95%CI: 0.76–9.15, P = 0.1508), and the median viral load for those who had it detectable was 36 (IQR: 25–67) copies/ml. No patient discontinued the study. There were no patients with virologic breakthrough throughout 48 weeks.

**Table 1:** Demographics and viro-immunological status of the population studied.

Parameter	Value
**Age in years, median (IQR)**	**45.6 (41.4–50.0)**
**Men, n (%)**	**32 (76.2)**
**Means of HIV-1 infection**	
**MsM (%)**	**16 (38.1)**
**HTSX (%)**	**9 (21.4)**
**IDU (%)**	**17 (40.5)**
**Years since diagnosis, years, median (IQR)**	**14 (10–21)**
**Smokers, n (%)**	**25 (59.5)**
**Prior AIDS, n (%)**	**27 (64.3)**
**HCV co-infection, n (%)**	**26 (61.9)**
**HCV treatment completed, n (%)**	**5 (31.2)**
**HBV co-infection, n (%)**	**1 (2.4)**
**Baseline CD4, median (IQR), cells/mm^3^**	**603 (389–901)**
**CD4 increase (IQR), cells/mm^3^**	**412 (258–573)**
**Nadir CD4 cell count <100 cells/mm^3^ (%)**	**14 (33.3)**
**Nadir CD4 cell count <200 cells/mm^3^ (%)**	**29 (69.0)**
**Baseline viral load, median (IQR), log_10_, copies/ml**	**1.28 (1.28–1.28)**
**Maximum viral load ≥5 log_10_, copies/ml (%)**	**26 (61.9)**
**Viral load decrease, median (IQR), log_10_, copies/ml**	**4.1 (3.6–4.7)**

**All values expressed as median (IQR  =  interquartile range) unless otherwise specified, MsM  =  men who have sex with men, HTSX  =  heterosexuals, IDU  =  intravenous drug users, AIDS  =  acquired immune deficiency syndrome, HCV  =  hepatitis C virus, HBV  =  hepatitis B virus, CD4 increase  =  current CD4-CD4 prior to starting of antiretroviral therapy, IQR  =  interquartile range.**

### Antiretroviral drug exposure

Most of the patients were highly antiretroviral-experienced with a median prior exposure to 5 (IQR: 3-6) different combination antiretroviral regimens. The d4T-based regimen was a salvage regimen for 62% of patients. Antiretroviral drug exposure is summarized in table 2.

**Table 2:** Antiretroviral drug exposure in the population studied.

Parameter	Value
**ART before switching**	
**IP-based, n (%)**	**27 (64.3)**
**NNRTI-based, n (%)**	**11 (26.2)**
**3 NRTIs, n (%)**	**2 (4.8)**
**IP+ NNRTI, n (%)**	**1 (2.4)**
**NRTI backbone before switching**	
**d4T+3TC, n (%)**	**10 (23.8)**
**d4T+TDF, n (%)**	**26 (61.9)**
**d4T+ddI, n (%)**	**3 (7.1)**
**d4T+ABC, n (%)**	**2 (4.8)**
**d4T alone, n (%)**	**1 (2.4)**
**No. of combination antiretroviral regimens, median (IQR)**	**5 (3–6)**
**HAART duration, m**	**127.5 (98.0–150.0)**
**Cumulated Individual drug exposure**	
**AZT exposure, m**	**12.0 (0.0–27.0)**
**AZT exposure, g**	**219.0 (0.0–492.1)**
**d4T exposure, m**	**89.0 (60.0–108.0)**
**d4T exposure, g**	**216.6 (146.0–262.8)**
**d4T exposure, mg/kg**	**1.046 (0.984–1.111)**
**ddI exposure, m**	**0.0 (0.0–21.0)**
**ddI exposure, g**	**0.0 (0.0–255.5)**
**ddC exposure, m**	**0.0 (0.0–0.0)**

**All parameters expressed as median and (interquartile range) unless otherwise specified. ART  =  Antiretroviral therapy, HAART  =  Highly active antiretroviral therapy, IP  =  protease inhibitor, NNRTI  =  non-nucleoside reverse transcriptase inhibitor, NRTI  =  nucleoside reverse transcriptase inhibitor, AZT  =  zidovudine, d4T  =  stavudine, ddI  =  didanosine, ddC  =  zalcitabine.**

### Metabolic and body composition parameters over time

Changes in metabolic and fat parameters are summarized in table 3. There were no statistically significant improvements in metabolic or insulin sensitivity parameters. There were no differences in the percentage of patients with total cholesterol ≥140 mg/dl at baseline and at week 48 (23.8% vs. 21.4%), or in LDL cholesterol ≥130 mg/dl (26.2% vs. 33.3%), HDL cholesterol ≤40 mg/dl (38.1% vs. 42.8%) and triglycerides ≥150 mg/dl (38.1% vs. 33.3%). Eleven patients (26.2%) were receiving lipid-lowering drugs at baseline and 16 (38.1%) at week 48 (P = 0.3500).

**Table 3:** Anthropometric, metabolic and fat parameters at baseline and 48 weeks after switching from stavudine to raltegravir.

Parameter	Baseline	48 weeks	P value
**Weight, kg**	71.0 (59.5-77.5)	68.5 (60.0-78.5)	**0.2626**
**BMI**	23.07 (21.08-25.69)	23.07 (20.69-25.71)	**0.1372**
**Waist circumference, cm**	86.0 (79.5-99.0)	89.0 (82.2-96.2)	**0.0464**
**WHR**	0.94 (0.90-0.99)	0.95 (0.89-1.00)	**0.3323**
**Systolic BP, mm Hg**	120 (110-129.5)	120 (110.7-125.2)	**0.9810**
**Diastolic BP, mm Hg**	74.0 (70.0-80.0)	77.0 (70.0-80.0)	**0.1386**
**Whole body fat, g**	13184 (8950-16691)	14941 (9430-18303)	**0.0027**
**Whole body fat, %**	18.6 (15.3-23.7)	21.7 (16.1-26.3)	**0.0037**
**Trunk fat, g**	8192 (5324-10.379)	8362 (5541-11057)	**0.0977**
**Left leg fat, g**	987 (794-1707)	1305 (861-1962)	**<0.0001**
**Limb fat mass, g**	3352 (2519-5827)	4247 (2848-6589)	**<0.0001**
**Trunk/limb fat ratio**	2.19 (1.68-2.99)	1.97 (1.49-2.88)	**0.0022**
**Fat mass ratio**	1.92 (1.42-2.56)	1.87 (1.29-2.48)	**0.0020**
**Fat mass index, kg/m^2^**	4.57 (3.08-5.64)	5.04 (3.19-6.47)	**0.0011**
**% left leg fat/BMI**	0.47 (0.36-0.65)	0.52 (0.40-0.80)	**<0.0001**
**Lean mass, g**	55895 (44837-61047)	54653 (45059-58274)	**0.1438**
**Lean mass/height^2^, kg/m^2^**	18.69 (16.17-20.42)	18.01 (16.12-19.96)	**0.9827**
**BMC, g**	2208 (1969-2525)	2216 (1963-2448)	**0.6035**
**BMD, g/cm^2^**	1.10 (1.04-1.19)	1.09 (1.03-1.18)	**0.2036**
**Metabolic syndrome, n (%)**	17 (40.5)	21 (50.0)	**0.5111**
**Total cholesterol, mg/dl**	191 (152-240)	196 (158-221)	**0.8216**
**Triglycerides, mg/dl**	171 (113-250)	203 (122-259)	**0.2245**
**HDL cholesterol, mg/dl**	44 (38-56)	41 (37-55)	**0.9643**
**Total cholesterol/HDL ratio**	4.30 (3.47-5.67)	4.56 (3.59-5.34)	**0.8776**
**LDL cholesterol, mg/dl**	98 (72-127)	105 (76-137)	**0.2194**
**Fasting glucose, mg/dl**	92 (86-97)	92 (86-97)	**0.2243**
**Fasting insulin, pmol/l**	54 (33-81)	37 (14-75)	**0.1614**
**HOMA-IR**	2.83 (1.66-4.20)	2.64 (1.54-4.78)	**0.1392**

**All parameters expressed as median and (interquartile range) unless otherwise specified. BMI  =  body mass index, WHR  =  waist-to-hip ratio, BP  =  blood pressure, BMC  =  Bone mineral content, BMD  =  Bone mineral density, HDL  =  High-density lipoprotein, LDL  =  Low-density lipoprotein, HOMA-IR  =  homeostasis model assessment method.**

All the patients had clinically-evident HALS. All the patients had lipoatrophy, but in addition 20 (47.6%) presented also with lipohypertrophy. No correlation between appendicular fat at baseline and prior exposure to d4T both in months and grams (r = 0.064, P = 0.6893) was found. Seventeen patients (40.5%) at baseline and 11 (26.2%) at week 48 had <3 kg of limb fat (OR = 1.92; 95%CI = 0.69-5.39, P = 0.2472). The median LSGS score at baseline was 8 (IQR: 8-12) and 8 (IQR: 7-10) at week 48 (P = 0.0070). The median facial score was 2 (IQR: 2-3) at 2 (IQR: 2-2) at week 48 (P = 0.0049). The median limb fat gain at 48 weeks was 800 grams (IQR: 270-1400). Whole body fat and limb fat mass increased significantly (Table 3). The median percent increase in limb fat mass was 20.9% (IQR: 7.4-33.6%). Twenty-two patients (52.4%) had an increase in limb fat ≥20% of baseline value. Indices of fat distribution symmetry showed significant improvements (Table 3). There was no correlation either between objective fat gain and LSGS score improvement (r = 0.109, P = 0.5211). No correlation between appendicular fat gain and prior exposure to d4T both in months and grams (r = 0.144, P = 0.3944). Trunk fat, lean mass, bone mineral content and bone mineral density did not change significantly.

A logistic regression analysis was performed taking increase in limb fat (< or ≥800 g) as the dependent variable and as independent all those baseline variables with a P<0.1 in univariate analyses (Table 4), and baseline weight. Baseline parameters independently associated with limb fat gain ≥800 grams were: baseline left leg fat (OR = 1.12; 95%CI: 1.00-1.25, P = 0.048, per 100 g) and length of HIV infection (OR = 0.83; 95%CI: 0.71-0.98, P = 0.023, per year).

**Table 4:** Baseline parameters significantly associated with limb fat gain (≥800 g limb fat) at week 48 on bivariate analysis.

Parameter	Limb fat gain <800 g (N = 21)	Limb fat ≥800 g (N = 21)	P value
**Age in years, median (IQR)**	47.0 (43.7-50.0)	43.0 (40.5-47.0)	**0.0321**
**Years since diagnosis, years**	13 (10-15)	7 (6-12)	**0.0105**
**HCV treatment completed, n (%)**	5 (23.8)	0 (0)	**0.0478**
**Baseline CD4, cells/mm^3^**	725 (434-935)	442 (367-699)	**0.0682**
**CD4 increase (IQR), cells/mm^3^**	534 (358-690)	355 (199-492)	**0.0296**
**Baseline viral load, log_10_, copies/ml**	1.28 (1.28-1.28)	1.28 (1.28-1.28)	**0.0449**
**No. of HAART regimens**	5 (4-7)	5 (2-8)	**0.0814**
**HAART duration, months**	131 (122.5-152.2)	98 (67.7-139.5)	**0.0870**
**Left leg fat, g**	881 (614-1298)	1131 (935-2024)	**0.0394**
**Limb fat, g**	2805 (2222-4618)	3738 (2928-5943)	**0.0719**
**Trunk/limb fat ratio**	2.39 (1.88-3.66)	1.91 (1.46-2.42)	**0.0761**
**HDL cholesterol, mg/dl**	45 (39-62)	40 (32-45)	**0.0761**
**HOMA-IR**	3.14 (2.40-12.38)	1.69 (1.47-2.93)	**0.0152**

**All values expressed as median (IQR  =  interquartile range) unless otherwise specified, MsM  =  men who have sex with men, HTSX  =  heterosexuals, IDU  =  intravenous drug users, AIDS  =  acquired immune deficiency syndrome, HCV  =  hepatitis C virus, CD4 increase  =  current CD4-CD4 prior to starting of antiretroviral therapy, IQR  =  interquartile range.**

**Variables not associated with limb fat gain ≥800 g with a P value <0.1 were: sex, means of HIV infection, smoking, prior AIDS, HCV co-infection, Nadir CD4 cell count <100 and <200 cells/mm^3^, maximum viral load >5 log_10_ copies/ml, ART before switching, NRTI backbone before switching, cumulated exposure for AZT, d4T, ddI and ddC, weight, BMI, waist circumference, WHR, systolic and diastolic BP, whole body fat, trunk fat, baseline limb fat <3 kg, fat mass ratio, fat mass index, percentage of left fat normalized by BMI, metabolic syndrome, total cholesterol, triglycerides, total cholesterol/HDL ratio, LDL cholesterol, fasting glucose, and fasting insulin.**

### SAT gene expression changes over time

**Figure 1:**
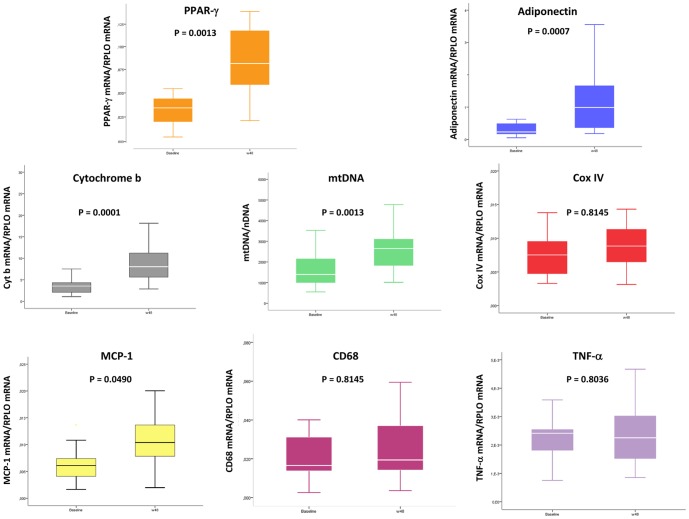
Changes in molecular markers in subcutaneous fat of patients with HALS switched from stavudine to raltegravir. Samples were retrieved at baseline and at week 48. Box plots show median, IQR and maximum and minimum values for each variable. mtDNA  =  mitochondrial DNA. PPAR gamma  =  peroxisome proliferator-activated receptor gamma. MCP-1  =  monocyte chemotactic protein 1. TNF alpha  =  tumor necrosis factor alpha. Cox IV  =  cytochrome oxidase subunit IV. mtDNA  =  mitochondrial DNA. nDNA  =  nuclear DNA.

Paired fat biopsies were available for 18 patients. Demographic, viro-immunological, metabolic and fat parameters of these patients were not different from the whole study group. PPAR gamma and adiponectin gene expression significantly increased from baseline (Figure 1). The relative abundance of mtDNA and Cyt b gene expression also significantly increased whereas Cox IV gene expression did not change significantly (Figure 1). TNF alpha and CD68 gene expression did not change significantly, although MCP-1 gene expression significantly increased (Figure 1). No correlation was found between limb fat gain and molecular markers change in SAT.

## Discussion

Our study shows that patients with long-lasting exposure to d4T, who have developed HALS, experienced a significant limb fat gain after switching to RAL. Consequently, indexes of body fat distribution symmetry also showed improvement after switching. Furthermore, fat gains were accompanied by improvements in gene markers of adipocyte differentiation and mitochondrial function at SAT level. Unfortunately, the objective fat gain did not translate into a benefit perceived by the patient, as occurs in other studies [4]. On the other hand, switching to RAL did not improve dyslipidemia and insulin sensitivity. Other body compartments such as lean mass, bone mineral content, and bone mineral density were unaffected by switching. No conclusion can be drawn from this study in terms of virologic efficacy of such a switch.

Switching from thymidine analogues to mitochondrially-friendly NRTI has been a popular strategy in recent years for lipoatrophy reversal. The rationale for this strategy is the well-known mitochondrial toxicity of thymidine analogues and the apparent lack of this toxicity of ABC and TDF [4], [5], [19], [20]. When d4T was switched to ABC or TDF, the increase in limb fat mass ranged from 329 to 493 grams after 48 weeks [4], [5]. The median amount of limb fat mass gain in the present study compares favorably to those reported in MITOX and RAVE trials [4], [5]. This can be due to the fact that RAL has no known toxic effects on mitochondria whereas ABC and TDF still exhibit some degree of mitochondrial toxicity. *In vitro* studies do not show a complete absence of toxicity on mitochondria for either ABC or TDF [21]. An *ex vivo* sub-study of ACTG 5224s showed a decrease in mtDNA fat content in patients on ABC- and TDF-based regimens [22], [23]. In the fat sub-study of the MONOI study, patients randomized to the boosted protease inhibitor (PI) monotherapy arm experienced a limb fat gain of 340 grams over 48 weeks, unlike what happened in the triple therapy control arm [24]. It is interesting to note that 79% of the NRTI discontinued in the MONOI study were ABC or TDF [24]. Furthermore, TDF toxicity is of mitochondrial etiology in other cell types such as renal proximal tubular cells [25]. Taken together, all these data suggest some low-level mitochondrial toxicity which in the event of long-lasting exposure may cause fat loss or may impede its recovery.

In our study two baseline variables were independently associated with a greater limb fat recovery, these being the length of HIV infection and baseline left leg fat. This suggests a concerted action of both the deleterious effects of HIV infection and prolonged exposure to antiretrovirals on the ability of SAT to recover. This is in line with the fact that HIV and antiretroviral drugs are able to affect biology and function not only of adipocytes but also of adipocyte precursor cells [26]-[28]. Furthermore, in patients exposed to AZT the length of exposure was an important variable determining the amount of baseline fat [29].

There have been two switching clinical trials with RAL [30], [31]. In both studies, patients on a stable boosted PI regimen had the PI changed for RAL, and such a switch was followed by significant improvement in lipid parameters in both studies, although fat was only studied in SPIRAL [32]. In this study there were no significant changes in the amount of limb fat between both arms [32]. However, it should be taken into account that the NRTI backbone was preserved throughout the study, and this included ABC or TDF in 77% of the patients.

We did not see any improvement in terms of dyslipidemia or insulin sensitivity. The use of d4T has been associated with hypercholesterolemia, hypertriglyceridemia and insulin resistance [20], [33]. The lack of improvement in metabolic parameters among our patients can be attributed to the fact that most of our patients (i.e. 64%) remained exposed to boosted PIs which are major contributors to the appearance of dyslipidemia and insulin resistance in HIV-infected patients [34].

The molecular findings of our study are in line with known drug side-effects and also with the observed fat changes. The well-known mitochondrial toxicity of d4T is exemplified by the significant increase in mtDNA and Cyt b, a subunit of the respiratory mitochondrial chain encoded by mtDNA [35]. On the other hand, the expression of Cox IV, which is encoded by nuclear DNA, was not significantly modified by switching. Both PI and NRTI exhibit deleterious effects on adipocyte biology and function, both in *in vitro* models and in *ex vivo* fat samples [35]. NRTI can impair adipocyte differentiation through down-regulation of PPAR gamma and C/EBP alpha [36], [37]. In addition, thymidine analogues also impair adiponectin adipocyte expression, an adipokine which sensitizes to insulin action and which has anti-inflammatory action [38]-[40]. In contrast, RAL has been reported to minimally affect adipocyte differentiation and metabolism, including unaltered adipogenic gene expression [41], [42]. Therefore, the improvement in PPAR gamma and adiponectin gene expression was expected after d4T withdrawal because of the neutral effect of RAL on the expression of these two genes. Furthermore, the present reported changes in adipose tissue gene expression are likely to be biologically and functionally relevant. Changes in the expression of PPAR gamma and PPAR gamma-responsive genes such as adiponectin have been significantly related to limb fat mass, further underscoring the importance of PPAR gamma expression in the development of lipoatrophy [36], [43].

Inflammation is a powerful force in the pathogenesis of HALS [1]. Data from the Lipostop trial suggest that d4T is directly involved in the generation of a pro-inflammatory environment in SAT of patients with HALS [44], and consequently d4T withdrawal was associated with a decrease in CD68 gene expression. This meant a decrease in macrophage infiltration at SAT level [44]. Our data are at odds with those of the Lipostop study since we did not observe significant changes neither in CD68 or in TNF alpha gene expression, and these changes occurred together with a significant increase in MCP-1 transcripts. The absence of improvement in the fat inflammatory status despite increased fat mass may be related to the persistence of systemic metabolic alterations since inflammation in SAT has been closely related to the induction of insulin resistance and dyslipidemia in metabolic pathologies such as obesity or congenital lipodystrophy [45].

Our study has inherent limitations. First, it is an observational study and therefore no causal relationship should or must be drawn. The lack of control arm is an important limitation of the study, but the evidence linking d4T exposure and HALS was so overwhelming when the study was designed, that having a control arm was not considered for ethical reasons [46]. Second, our study is of limited duration, but its time span was sufficient to show a significant improvement in limb fat and in SAT molecular parameters. Alternatively there could be differences in cell populations not reflected by differences in gene transcripts. The evolving trend of limb fat beyond 48 weeks would probably not be so pronounced in terms of gain, but we can only speculate about that. Third, our sample size is small and this may prevent achieving statistically significant results for some variables that show a trend to significance. Fourth, the clinical significance of switching from d4T to RAL is of limited usefulness in resource-rich settings and even in those with limited resources, but our study demonstrates that it can be useful for those cases that may exist.

In summary, switching from d4T to RAL in patients with HALS induces a remarkable increase in limb fat mass with and improvement of molecular markers of adipocyte differentiation and mitochondrial function in SAT, without beneficial effects on dyslipidemia or insulin sensitivity.
